# Analysis of the Association Between the Number of Intensivists and the Use of Cardiovascular Agonists: An Ecological Study Using Data From National Databases of Japan

**DOI:** 10.7759/cureus.48912

**Published:** 2023-11-16

**Authors:** Shohei Ono, Keiki Shimizu

**Affiliations:** 1 Anesthesiology and Critical Care, Jichi Medical University Saitama Medical Center, Saitama, JPN; 2 Emergency and Critical Care Center, Tokyo Metropolitan Tama Medical Center, Tokyo, JPN

**Keywords:** national database open data, dopamine, vasoconstrictor, inotrope, intensivists

## Abstract

Background

Previous studies have demonstrated a correlation between management by intensivists and a decrease in hospital stay and mortality, yet the underlying reason remains unknown. Using open data from the National Database of Health Insurance Claims and Specific Health Checkups of Japan (NDB) and other databses, the present study aimed to explore the relationship between inotrope and vasoconstrictor use and the number of intensivists.

Materials and methods

Cardiovascular agonists listed in the 2020 NDB for which the total dose was known were included for analysis. Trends in cardiovascular agonist use over six years were then graphically assessed, and a linear regression model with the use of each target drug per prefecture as the objective variable in the 2020 data was created to analyze the impact of intensivists on drug use.

Results

A total of 61 drugs were classified into eight groups based on their composition, and drug use in each of the 47 prefectures was tabulated. Both the rate of use and cost showed a yearly decrease for dopamine but a yearly increase for norepinephrine. Multivariable analysis indicated that the number of intensivists was only significant for dopamine, which had a coefficient of -310 (95% CI: -548 to -72, p = 0.01) but that no such trend was evident for the other drugs.

Conclusions

The results demonstrated that an increasing number of intensivists in each prefecture correlated with decreasing use of dopamine, possibly explaining the improved outcomes observed in closed ICUs led by intensivists. Further research is warranted to establish causality.

## Introduction

Management practices within intensive care units (ICU) are highly dependent on the local medical system and lack standardization. Nevertheless, several studies have highlighted the importance of ICU management by intensivists in decreasing the length of hospital stay and mortality. A study of 10,900 patients in 34 ICUs at 27 hospitals demonstrated that active involvement by intensivists resulted in a reduction of medically cost-weighted hospital days [1]. A systematic review of 26 observational studies found that mandatory intensivist consultation or closed ICUs, which are all managed by intensivists, reduced hospital mortality by 29% compared to low-intensity groups with no intensivist or elective intensivist consultation [2]. The superiority of ICU management by intensivists has also been demonstrated in several studies comparing closed ICUs led by intensivists to open ICUs managed by general physicians [3-9]. A meta-analysis of five randomized controlled trials enrolling 6160 participants between 1992 and 2007 found that the mortality rate was 31% higher in open ICUs than in closed ICUs (OR 1.31; 95%CI 1.17-1.48; p < .001) [10].

While the importance of intensivists is undeniable, it has yet to be determined how their activities affect patient prognosis. An earlier study found that closed ICUs managed by intensivists had a higher rate of deep vein thrombosis prophylaxis, earlier initiation of enteral nutrition [4], and greater adherence to guidelines [3]. However, these findings only accounted for a limited aspect of ICU treatment, and no reports exist on the medications used. Thus, the present study aimed to evaluate trends in drug selection by intensivists by focusing specifically on the use of inotropes and vasoconstrictors commonly used in ICUs.

In the ICU, inotropes and vasoconstrictors are frequently used in patients with shock either cardiogenic or distributive; thus, the present study refers to these agents as cardiovascular agonists. Cardiovascular agonists exist in a range of forms and combinations, and their use varies among physicians. Vasoconstrictors are used for patients with hypotension, including those with septic shock. A systematic review of 28 randomized controlled trials demonstrated that dopamine may increase the risk of arrhythmia and mortality significantly more than norepinephrine, but the evidence of differences among the remaining six vasoconstrictors studied was insufficient [11]. Epinephrine, norepinephrine, and dopamine are sometimes referred to as inoconstrictors because they stimulate beta-receptors whereas vasopressin functions purely as a vasoconstrictor without any inotropic effects. Currently, norepinephrine is the most widely used for shock, and no other vasoconstrictors have demonstrated superiority to norepinephrine in clinical trials [12]. Vasopressin and epinephrine, however, serve as secondary options for shock treatment [11,12]. Dopamine use has declined owing to recent clinical trials questioning its efficacy [13]. Dobutamine and milrinone, which are classified as inodilators because they have beta-stimulating and vasodilating effects, are primarily used for cardiogenic shock. Despite potentially improving circulatory failure in patients with poor cardiac function, their vasodilatory properties may militate against increasing blood pressure, and the evidence supporting their use is weaker than the evidence supporting vasoconstrictor use [14].

While these agents are commonly used within the ICU to treat circulatory failure, previous studies have shown that heterogeneity in the patient background and variability in physician behavior results in significant variability in their use [12,15-19]. Information about factors contributing to the variability in physicians’ choice of drugs is still scarce. The present ecological study based on the National Database of Health Insurance Claims and Specific Health Checkups of Japan (NDB) open data aimed to examine the impact of the number of intensivists in a given region on the rate of cardiovascular agonist use.

## Materials and methods

Study design

The present ecological study on the association between the use of cardiovascular agonists and the distribution of intensivists in 47 Japanese prefectures was based on open data from the NDB open data [20] and other databases. The NDB is a comprehensive, national, administrative claims database for health insurance and health examinations and is curated by the Ministry of Health, Labour and Welfare (MHLW). The NDB open data were generated by aggregating portions of the NDB that do not contain confidential information. Consequently, researchers using NDB open data do not have access to patient- or facility-level information. This study consisted of two parts, both of which used NDB open data: (i) longitudinal analysis of the number of intensivists and (ii) the use of each cardiovascular agonist over time. The study complied with the principles of the Declaration of Helsinki. Ethical review and informed consent were waived as all the data used in the analysis were anonymized and did not include individual patient information.

The present study included drugs listed in the seventh, most recent, update of the NDB (April 1, 2020, to March 31, 2021) as inotropes, vasoconstrictors, or drugs that were primarily used as a cardiovascular agonist. Drugs that were not consistently administered as a cardiovascular agonist and drug entries without information on the total dose were excluded. Then, two researchers reviewed those drugs to determine the appropriate classification of cardiovascular agonists. Based on this classification, we tabulated the total use and cost (Japanese Yen) in Japan for each drug in the second (April 1, 2015 to March 31, 2016), third (April 1, 2016 to March 31, 2017), fourth (April 1, 2017 to March 31, 2018), fifth (April 1, 2018 to March 31, 2019), sixth (April 1, 2019 to March 31, 2020), and seventh database updates. Owing to differences in the analysis method, the first update was excluded. The second part comprised a cross-sectional analysis of target drug use per 47 prefectures in the seventh NDB open data update. Thus, the number of individual data in this analysis was 47.

Data collection

NDB open data contain annual figures for the use of injectable drugs per prefecture. The data lists the 100 most frequently prescribed injectable drugs by indication and excludes drugs with a low frequency of use. We calculated the total use and cost in Japan for each drug from the second to the seventh update based on the classification we created by the seventh update. Based on the same classification, we tabulated the total use and cost (in (Japanese Yen) per prefecture for each drug in the seventh database update without regard to the quantity of the components or formulation (vial, ampule, etc.). We also collected the medical and geographic features of each prefecture that might be linked to disparities in the use of cardiovascular agonists. The number of intensivists, the number of patients with central venous catheter (CVC) placement, the cumulative number of days of CVC placement (CVC days), the number of patients receiving invasive mechanical ventilation (IMV) > five days, and the number of physicians were included as medical features.

The most recent data on the number of intensivists (April 1, 2022) were collated manually from information published on the website of the Japanese Society of Intensive Care Medicine (JSICM) [21]. The JSICM was requested to provide past data on the total number of intensivists (April 1, 1998, to April 1, 2022), but data for individual prefectures were unavailable. Information about CVC placement, CVC days, and IMV was obtained from the injections and procedures table of the seventh update of the NDB open data. The number of physicians was obtained from the 2020 Summary of Statistics on Physicians, Dentists, and Pharmacists published by the MHLW [22]. Population, population density, proportion of elderly individuals (≥ 65 years old), proportion of male individuals, area, and mean age were included as geographic features. Data on population, the proportion of elderly individuals, the proportion of male individuals, and mean age were obtained from the Summary of Population Estimation Results published by the Ministry of Internal Affairs and Communications [23]. Data on the area were obtained from the Geographical Survey Institute of the Ministry of Land, Infrastructure, Transport and Tourism [24]. The population density was calculated by dividing the population by area. Data on population were expressed as per 100,000 population.

Statistical analysis

In the first part, we visually analyzed trends in the use of the target drugs and the associated cost from the second to the seventh update of the NDB open data by graphically depicting the trend in the use of each cardiovascular agonist and the number of intensivists. In the second part, we divided the intensivists into two groups by median variable, and the characteristics of the prefectures and target drug use were compared with Student's t-test for variables with a normal distribution or Wilcoxon's rank sum test for variables with a non-normal distribution. To assess the relationship between the number of intensivists and drug use, scatter plots, box plots, and heat maps of Japan were used. Next, we performed linear regression with drug use as a dependent variable and the number of intensivists as an independent variable in univariable analysis and multivariable analysis adjusting for population, population density, CVC days, and physician number. These analytical models were constructed for each drug category using population-standardized numbers converted to per 100,000 population as objective variables. Instances where the total dose was known but information about the dose per prefecture was unavailable were included in the analysis using the mean assignment method. The importance of intensivists was assessed from different perspectives; first, by comparing it to the statistical significance of other independent variables on multivariable analysis; then by developing the multivariable analysis into a machine learning model to assess the importance of the features (Random Forest, Neural network, eXtreme Gradient Boosting, Lasso regression). Ten cross-validations were performed for each of the four hyperparameter patterns to create the machine-learning model.

Several sensitivity analyses we performed to verify the robustness of the results. First, multivariable analysis was repeated with cost (in Japanese Yen) instead of drug use as the dependent variable. Second, zero imputation was performed for missing values instead of using the mean assignment method. Finally, the stepwise method was employed to select and analyze combinations of independent variables in the multivariable analysis.

The data were expressed as the mean and standard deviation or the median and interquartile range (IQR) as appropriate. The two-sided significance level was set at p=0.05, and all statistical analyses were performed using R-4.2.1 (R Foundation for Statistical Computing, Vienna, Austria).

## Results

**Figure 1:**
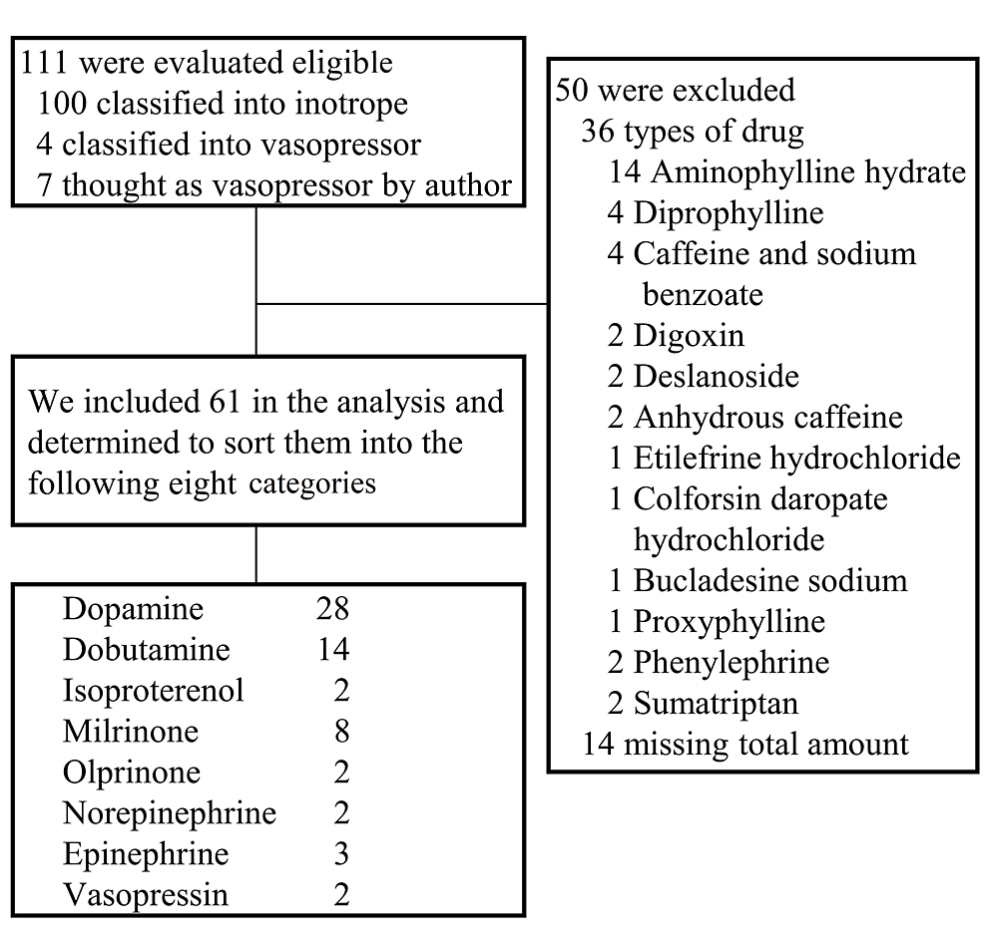
Flowchart of classification of the cardiovascular agonists listed in the 2020 NDB open data NDB: National Database of Health Insurance Claims and Specific Health Checkups of Japan

**Figure 2:**
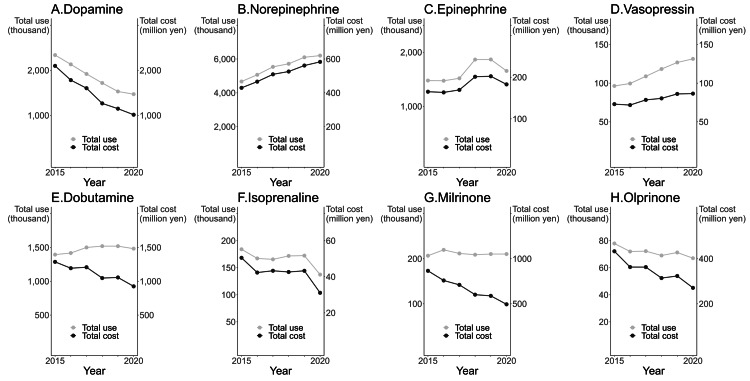
Cardiovascular agonist use and cost (in Japanese Yen) by year in each category Owing to differences in the analysis method, the first update of the NDB was excluded. NDB: National Database of Health Insurance Claims and Specific Health Checkups of Japan

**Figure 3:**
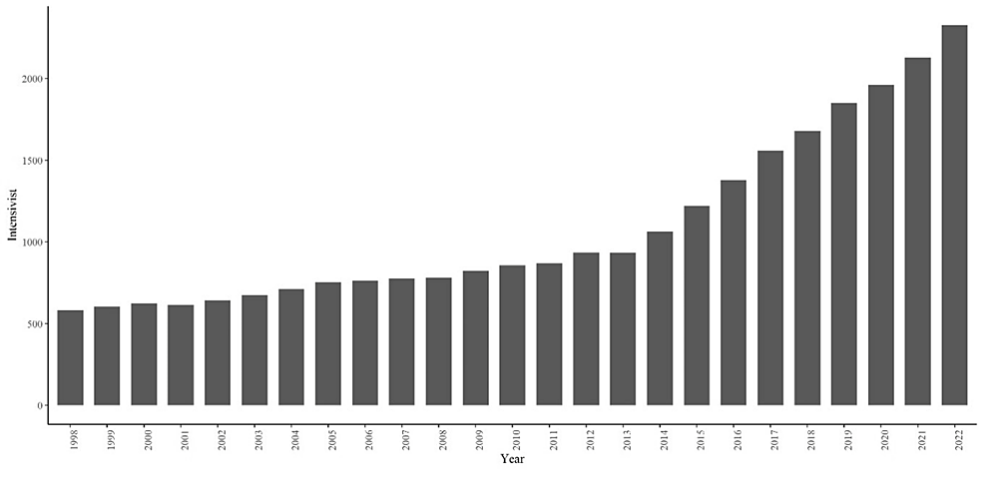
Number of intensivists certified by the Japanese Society of Intensive Care Medicine over the years

A total of 61 drugs were categorized into eight groups based on their composition (Figure 1). The rate of use and cost of each cardiovascular agonist were tabulated by year based on this classification to visualize the trends (Figure 2). Both the rate of use and cost declined annually for dopamine but increased yearly for norepinephrine. No trend was observed for the other drugs. The number of intensivists rose annually, demonstrating that the trend in the number of intensivists was inversely proportional to dopamine use and positively proportional to norepinephrine and vasopressin use (Figure 3).

**Figure 4:**
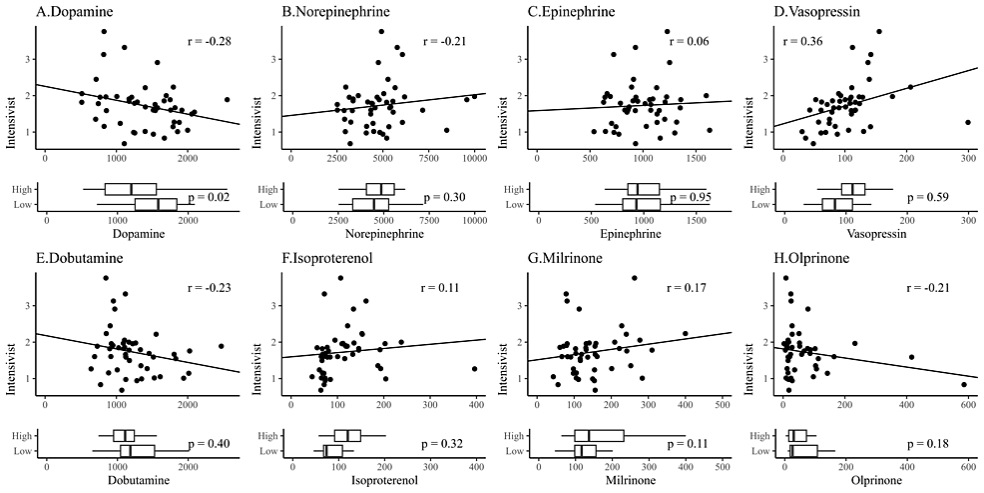
Scatter and box plots showing the correlation between cardiovascular agonist use and the number of intensivists The number of intensivists and cardiovascular agonist use were expressed as the rate per 100,000 population. Furthermore, the number of intensivists in the boxplots was transformed into a binary variable bounded by the median value of 1.69

**Figure 5:**
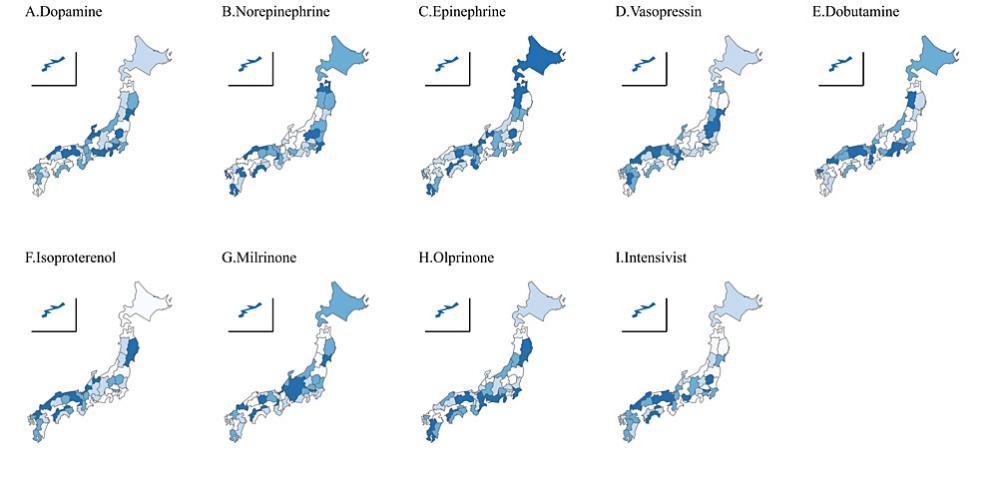
Heat map of Japan visualizing the distribution of cardiovascular agonist use and the number of intensivists Increasing darkness of hue signifies increasing frequency of use and increasing number of intensivists. For drugs with a negative correlation coefficient with the number of intensivists (dopamine, dobutamine, and olprinone), darker hues signify lower frequencies of use.

We then collected data for each of the 47 prefectures. Drug use was tabulated for the seventh NDB open data update. We found an average of 27 missing values for each drug, which we substituted with the mean assignment method. Table 1 shows the geographic features, medical features, and cardiovascular agonist use for the two prefectural groups divided by the median number (1.69) of intensivists. The findings indicated a statistically significant difference in population density (p = 0.009), mean age (p = 0.002), and proportion of elderly individuals (p = 0.006) among the prefecture features. No statistically significant difference was found in the medical features between the groups. In terms of the rate of drug use, dopamine use was statistically lower in prefectures with a larger number of intensivists (p = 0.02), but no statistically significant difference was found for the other medications. Next, the relationship between the number of intensivists and drug use was demonstrated using a scatter plot and box plot with the number of intensivists treated as a binary variable (Figure 4). As shown in Table 1, dopamine was the only drug whose use differed significantly between the two groups, which is confirmed visually by the box plot. The scatter plot revealed a negative correlation between dopamine and the number of intensivists, which had the second-highest absolute correlation coefficient (r = -0.28) after vasopressin (R = 0.36). A heat map of Japan demonstrated that regions with more intensivists tended to have lower dopamine use. No similar trend was observed for the other drugs (Figure 5).

**Table 1:** Baseline prefectural characteristics divided by the median number (1.69) of intensivists For medical characteristics and cardiovascular agonist use, variables were expressed as population-standardized values converted per 100,000 population. For geographic characteristics, variables were expressed as is.

	Fewer intensivists (N=23)	More intensivists (N=24)	p-value
Geographic features			
Population, median (IQR)	1238 (998, 1959)	1911 (1338, 5218)	0.06
Area (km^2^), median (IQR)	6097 (4217, 10133)	5643 (3936, 7156)	0.18
Population density (/km^2^), median (IQR)	186 (136, 306)	341 (210, 745)	0.009
Mean age (years), mean (SD)	49 (1)	48 (2)	0.002
Proportion of males (%), mean (SD)	48 (1)	48 (1)	0.62
Proportion of elderly (%), mean (SD)	32 (2)	30 (3)	0.006
Medical features			
Physicians, mean (SD)	250 (44)	273 (35)	0.05
Invasive mechanical ventilation, mean (SD)	6470 (1988)	6483 (2278)	0.98
Central venous catheter days, mean (SD)	9217 (2996)	8353 (1921)	0.24
Central venous catheter placement, mean (SD)	374 (87)	354 (71)	0.38
Cardiovascular agonist use			
Dopamine, mean (SD)	1534 (410)	1225 (481)	0.02
Norepinephrine, mean (SD)	4546 (1432)	5043 (1772)	0.30
Epinephrine, mean (SD)	994 (267)	990 (245)	0.95
Vasopressin, mean (SD)	91 (54)	114 (35)	0.10
Dobutamine, mean (SD)	1278 (388)	1182 (387)	0.40
Isoproterenol, mean (SD)	106 (77)	125 (45)	0.32
Milrinone, mean (SD)	131 (57)	167 (89)	0.11
Olprinone, mean (SD)	90 (140)	47 (50)	0.17

Finally, generalized linear regression analysis was conducted to assess the relationship between the number of intensivists and the rate of drug use. Univariable and multivariable models were created with the rate of use of eight drugs as the objective variable, and the coefficients for the number of intensivists as an explanatory variable were estimated for each model (Table 2). On univariable analysis, the coefficient of the number of intensivists was not statistically significant in either model. On multivariable analysis, the number of intensivists was statistically significant (coefficient: -310; 95%CI -548 to -72; p = 0.01) only in the model with dopamine as the objective variable, indicating that dopamine use declined as the number of intensivists increased. No statistically significant difference was found for the other models.

**Table 2:** Estimated coefficients of the number of intensivists in linear regression predicting cardiovascular agonist use The multivariable analysis was adjusted for population density, the number of physicians, invasive mechanical ventilation, and area.

Objective variables	Non-adjusted OR (95% CI)	p-value		Adjusted OR (95% CI)	p-value
Dopamine	-211 (-419 to -3)	0.0		-310 (-548 to -72)	0.01
Norepinephrine	372 (-368 to 1112)	0.33		378 (-536 to 1293)	0.42
Epinephrine	23 (-94 to 140)	0.70		-29 (-169 to 111)	0.69
Vasopressin	26 (6 to 46)	0.01		20 (-5 to 45)	0.12
Dobutamine	-139 (-313 to 36)	0.13		-115 (-336 to 105)	0.31
Isoproterenol	11 (-18 to 40)	0.44		-14 (-48 to 20)	0.43
Milrinone	21 (-14 to 56)	0.25		31 (-12 to 75)	0.16
Olprinone	-35 (-82 to 13)	0.16		-49 (-109 to 11)	0.12

Further, the number of intensivists was compared with the other variables in terms of their impact on dopamine use. On the multivariable analysis of the dopamine model, no independent variables except the number of intensivists differed significantly (Table 3). A machine learning model was then created to assess the influence of the number of intensivists on dopamine use (Table 4). The machine models with the assumption of linearity (Linear and Lasso Regression) performed better than the other models (Random Forest, eXtreme Gradient Boosting, and Neural Network); the number of intensivists was the most important feature in the regression models.

**Table 3:** Estimated coefficient and p-value of independent variables in multivariable analysis IMV: invasive mechanical ventilation

	Coefficient (95% CI)	p-value
Intensivists, /100,000	-310 (-548 to -72)	0.01
Population density, /km^2^	-0.08 (-0.2 to 0.04)	0.19
Physicians, /100,000	2.71 (-1.63 to 7.04)	0.23
Area, km^2^	0 (-0.01 to 0.01)	0.69
IMV, /100,000	0.05[-0.03 to 0.12]	0.27

**Table 4:** Performance of machine learning models and the importance of the number of intensivists IMV: invasive mechanical ventilation; NA: not applicable

	Random forest	Lasso regression	eXtreme Gradient Boosting	Neural network	Linear regression
Performance					
Root mean squared error	427	369	504	1436	428
Decision coefficient	0.258	0.568	0.31	0.299	0.493
Mean absolute error	368	320	419	1365	367
Feature importance					
Intensivist	60	100	55	33	100
Population	47	86	0	100	NA
Population density	73	0	16	92	43
Mean age	100	0	6	65	NA
Proportion of elderly persons	97	67	100	78	NA
Area	20	0	3	11	0
Proportion of physicians	0	30	0	0	38
IMV	47	52	14	31	34
CV days	47	98	12	5	NA
CV placement	41	0	16	11	NA

Our study also involved several sensitivity analyses. An analytical model was created with the cost of the drugs treated as the dependent variable (Table 5). Univariable analysis indicated that the number of intensivists was not statistically significant in either model. However, when the cost of dopamine was included as an objective variable in multivariable analysis, the number of intensivists emerged as statistically significant (coefficient -192855; 95%CI -375394 to -10316; p = 0.05). In contrast, the number of intensivists was not statistically significant in the other models. Next, missing value analysis was performed by substituting zero for missing drug use data (Table 6). The results of both univariable (coefficient: -223; 95%CI: -430 to -17; p = 0.04) and multivariable (coefficient: -302; 95%CI: -549 to -55; p = 0.02) analyses indicated that the number of intensivists was statistically significant in the dopamine model. The number of intensivists in the vasopressin models was statistically significant on univariable analysis (coefficient: 28; 95%CI: 7 to 49; p = 0.01), but this significance was not observed on multivariable analysis (coefficient: 22; 95%CI: -4 to 49; p = 0.11). Finally, the stepwise approach was used, with intensivists, mean age, CVC days, and the proportion of elderly individuals as the explanatory variables in multivariable analysis (Table 7). The number of intensivists was once again found to be statistically significant (coefficient: -222; 95%CI: -402 to -37; p = 0.02).

**Table 5:** Sensitivity analysis: estimated coefficients for the number of intensivists in linear regression predicting the cost (Japanese Yen) of each cardiovascular agonist The multivariable analysis was adjusted for population density, the number of physicians, invasive mechanical ventilation, and area.

Objective variables	Non-adjusted OR (95%CI)	p-value		Adjusted OR (95%CI)	p-value
Dopamine	-131328 (-287049 to 24393)	0.11		-192855 (-375394 to -10316)	0.05
Norepinephrine	34966 (-34599 to 104531)	0.33		35569 (-50394 to 121531)	0.42
Epinephrine	418179 (-261235 to 1097594)	0.23		195029 (-135603 to 525662)	0.25
Vasopressin	14854 (3503 to 26206)	0.01		11468 (-2689 to 25624)	0.12
Dobutamine	-45262 (-147080 to 56555)	0.39		-7133 (-136258 to 121992)	0.91
Isoproterenol	2554 (-3875 to 8982)	0.44		-3347 (-10822 to 4128)	0.39
Milrinone	53405 (-29655 to 136465)	0.21		54693 (-50752 to 160138)	0.32
Olprinone	-140985 (-337148 to 55178)	0.17		-201736 (-447490 to 44018)	0.12

**Table 6:** Sensitivity analysis: estimated coefficients for the number of intensivists in linear regression using zero imputation The multivariable analysis was adjusted for population density, the number of physicians, invasive mechanical ventilation, and area.

Objective variables	Non-adjusted OR (95%CI)	p-value		Adjusted OR (95%CI)	p-value
Dopamine	-223 (-430 to -17)	0.04		-302 (-549 to -55)	0.02
Norepinephrine	371 (-369 to 1111)	0.33		379 (-535 to 1294)	0.42
Epinephrine	-1 (-3 to 2)	0.57		0 (-2 to 2)	0.81
Vasopressin	28 (7 to 49)	0.01		22 (-4 to 49)	0.11
Dobutamine	-139 (-317 to 39)	0.13		-106 (-333 to 120)	0.36
Isoproterenol	11 (-17 to 40)	0.44		-13 (-48 to 21)	0.45
Milrinone	20 (-20 to 60)	0.34		38 (-9 to 84)	0.12
Olprinone	-38 (-87 to 11)	0.14		-51 (-113 to 11)	0.12

**Table 7:** Sensitivity analysis: estimated coefficients for the number of intensivists in multivariable analyses using the stepwise approach The stepwise approach was used to select the number of intensivists, central venous catheter insertion, mean age, and proportion of elderly individuals.

Objective variables	Adjusted OR (95%CI)	p-value
Dopamine	-222 (-407 to -37)	0.02
Norepinephrine	302 (-483 to 1088)	0.46
Epinephrine	61 (-49 to 171)	0.28
Vasopressin	19 (-1 to 39)	0.07
Dobutamine	-123 (-314 to 68)	0.22
Isoproterenol	3 (-27 to 33)	0.84
Milrinone	20 (-18 to 58)	0.31
Olprinone	-21 (-71 to 30)	0.42

## Discussion

Previous studies of intensivist-led ICU management are scarce, making the present ecological study the first to reveal a correlation between the number of intensivists and drug use. A significant decrease in dopamine use was noted over time, a trend that was not replicated with the other medications. Meanwhile, there was an increase in the number of intensivists, and a negative correlation was detected between dopamine use and the number of intensivists in terms both of overall duration of use and of individual prefectures. These results were consistent across multiple sensitivity analyses and were deemed to be robust. The significance of the number of intensivists was highlighted by the results of the machine learning model, which found that prefectures with a large number of intensivists tended to have a low rate of dopamine use, indicating that the number of intensivists had a pronounced impact on dopamine use.

Our study had two key clinical implications. First, the results highlighted the factors influencing dopamine use in clinical practice. Owing to its lack of efficacy in numerous clinical trials [13,25-27], dopamine has been discontinued in many countries, as the Surviving Sepsis Campaign Guidelines’ recommendation against its use for renal protection shows [28-30]. However, the present study found that dopamine is still being used in Japan, and multivariable analysis determined that, in a given prefecture, as the number of intensivists increased, dopamine use decreased. Conversely, other potential explanatory variables such as population density and number of physicians were found to be statistically non-significant. Second, our study highlighted the trend toward intensivist-led critical care management. Previous studies have established that closed ICUs managed by intensivists tend to produce better outcomes [3-9] although the reason for this was unclear. Our study highlighted the medications that intensivists tend to prescribe, thus providing a possible explanation for the superior outcomes observed in closed ICUs led by intensivists. The inappropriate use of drugs remains a crucial but unresolved clinical issue; our findings suggest that increasing the number of intensivists worldwide might lead to more appropriate use of cardiovascular agonists.

The present study has three salient strengths. First, it employed comprehensive census data from Japan while the majority of previous studies investigating the significance of intensivists focused on smaller samples derived from individual hospitals. Second, the study's findings were clear, as multiple sensitivity analyses produced congruent outcomes. Last, the present study assessed the number of intensivists instead of examining the difference between closed and open ICUs, thereby demonstrating the direct benefit of increasing the number of intensivists worldwide.

The present study has three serious limitations. First, its cross-sectional design precludes any conclusions regarding a causal relationship between the increased number of intensivists and decreased dopamine use. Second, there may have been unmeasured confounders, as the data used in this study were regional rather than individual or hospital-based. Third, the restriction of the scope of this study to Japan raises questions about the generalizability of the results to other countries. Longitudinal studies incorporating individual data from multiple nations are therefore warranted.

## Conclusions

This study indicated a significant decrease in dopamine use in regions with a higher concentration of intensivists. As this study was cross-sectional, further research is required to establish a causal link. An increase in the number of intensivists might lead to standardization of care; if so, the result would further emphasize the need for more intensivists worldwide. Given the cost and effort involved in producing more intensivists, it is important to clarify their relationship not only to dopamine use but also to other aspects of medicine.
